# Carcass assessment and value in the Australian beef and sheepmeat industry

**DOI:** 10.1093/af/vfae005

**Published:** 2024-04-16

**Authors:** Sarah M Stewart, Rod Polkinghorne, David W Pethick, Liselotte Pannier

**Affiliations:** School of Agriculture, Centre for Animal Production and Health, Food Futures Institute, Murdoch University, Perth 6150, Australia; Birkenwood Pty. Ltd., Murrurundi, NSW 2338, Australia; School of Agriculture, Centre for Animal Production and Health, Food Futures Institute, Murdoch University, Perth 6150, Australia; School of Agriculture, Centre for Animal Production and Health, Food Futures Institute, Murdoch University, Perth 6150, Australia

**Keywords:** grading, meat quality, objective measurement, technology, value, yield

**Implications**

- The Australian beef and lamb industries have shifted from utilizing a carcass classification system to consumer-focused carcass grading, through the Meat Standards Australia grading system.
- Grading systems must be dynamic to meet changing consumer preferences.
- Objective technologies can increase the accuracy and precision of yield and eating quality measurement inputs into grading models.
- Combining carcass yield estimates with consumer value can underpin transparent value-based trading systems.
- Value-based trading can generate significant industry benefits by delivering the desired product to the consumer, improving processing efficiency and optimization, and providing more precise carcass feedback and market signals to livestock suppliers.

## Introduction

Beef and sheepmeat are major high-volume foods produced in Australia for both domestic consumption and export markets. Australian carcass and meat evaluation has evolved from very basic and imprecise carcass assessments to more sophisticated systems over the last 30 years, reflecting changing technologies, market systems, and consumer preferences, with significant investment in eating quality research (Polkinghorne, 2018).

Uniquely to Australia, systems of carcass classification and description largely based on sex, dentition, and crude measures of yield (carcass weight and fat depth) have expanded with the introduction of consumer-focused grading through the Australian Meat Standards Australia (**MSA**) system for beef and sheepmeat. The MSA grading system aims to deliver an eating quality guarantee to consumers through the implementation of eating quality grades (Polkinghorne and Thompson, 2010; Pannier et al., 2018) based on a combination of carcass classification and grading evaluation traits as defined under the AUS-MEAT language (AUS-MEAT, 2022).

Most recently, the development and commercialization of new objective grading technologies have provided dramatically improved yield predictions and assisted quality grading trait accuracy and repeatability (Gardner et al., 2021b). To maximize carcass value across the supply chain, accurate carcass grading systems for both eating quality and yield are paramount (Pethick et al., 2021). Therefore, objective grading technologies to accurately measure lean meat yield (**LMY**) and eating quality traits will be critical to implement value-based payment systems in the Australian beef and sheepmeat industries.

This paper addresses the current grading systems utilized in Australia with the potential utilization of combining yield and eating quality systems in value-based payment systems that can accelerate industry efficiency and profitability through consumer pricing signals.

## Beef and Lamb Carcass Classification and Grading in Australia

### AUS-MEAT carcass description and chiller assessment

In Australia, AUS-MEAT Limited is the formal standards body with government delegation responsible for defining export market product description and management of industry standards and verification for trade description. AUS-MEAT is an industry-owned not-for-profit company established between Meat and Livestock Australia and the Australian Meat Processor Corporation which manages industry quality standards through the AUS-MEAT chiller assessment language and national accreditation standards. Both are designed to protect the integrity of the AUS-MEAT language and Australian industry related to sale, distribution, and export of Australian red meat.

The AUS-MEAT language uses descriptive terms to describe meat products to meet market requirements both nationally and internationally. This language forms the basis of the national description system of carcass measurements. Itis utilized for the classification of Australian meat and livestock and is also internationally recognized through the United Nations Economic Commission for Europe. Proposed language changes and approval and implementation of changes to AUS-MEAT language are progressed through a consultative process with industry stakeholders and the Australian Meat Industry Language and Standards Committee.

**Figure 1.**
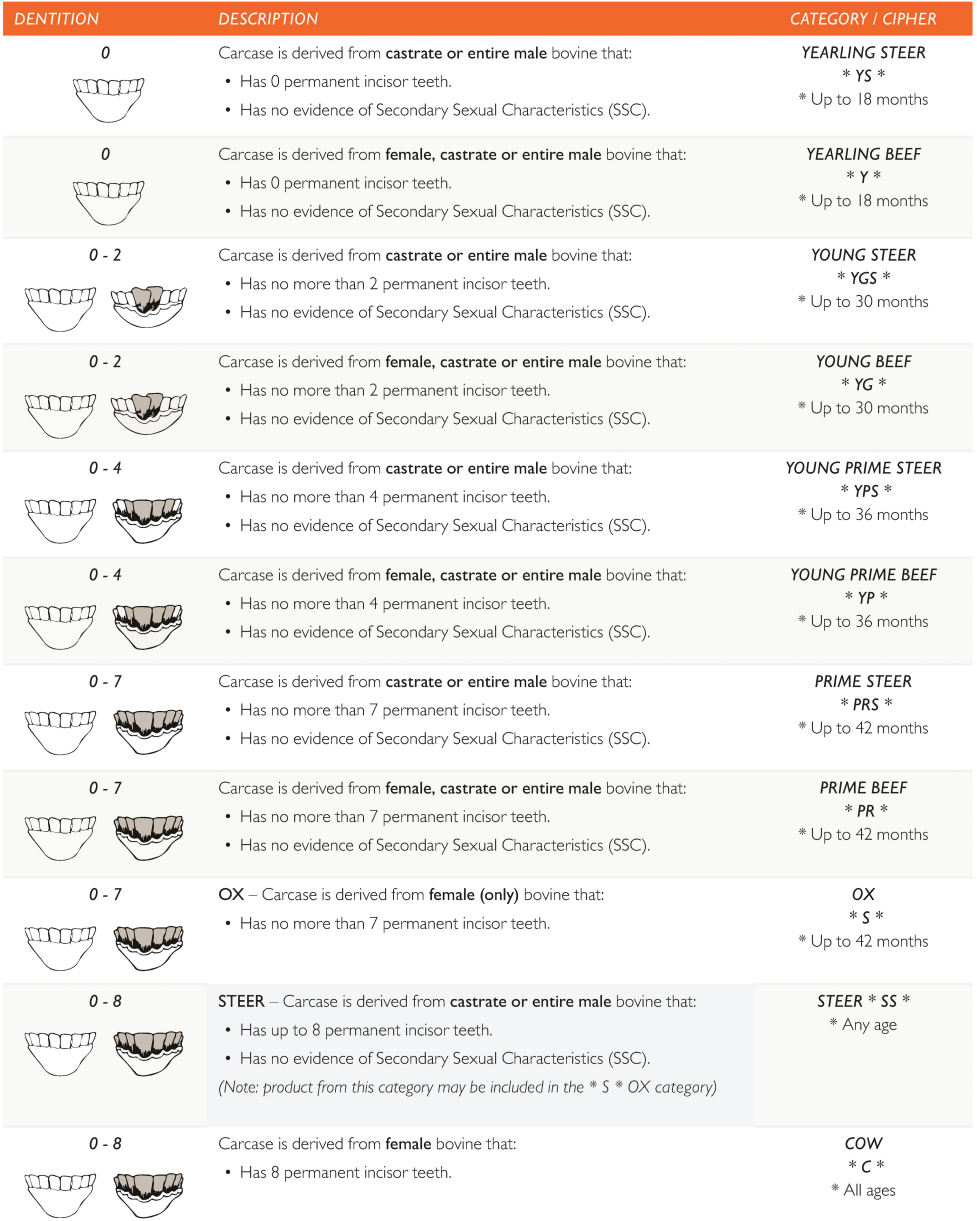
Bovine categories based on sex and dentition classes (AUS-MEAT, 2022).

**Figure 2.**
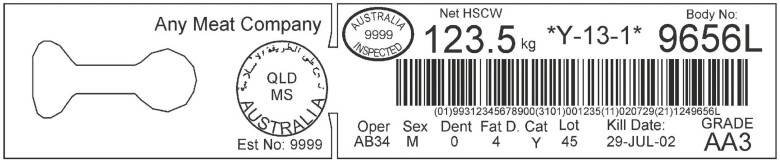
An example of a beef carcass ticket (AUS-MEAT, 2022).

Detailed language description can be accessed through the Handbook of Australian Meat (AUS-MEAT, 2022). When the AUS-MEAT language was first established, dentition was included as a proxy for animal age due to associated changes in meat tenderness. Accordingly, dentition in conjunction with sex provided a framework for the description of beef and lamb carcass categories which are used as trade ciphers on carcass tickets, feedback sheets, and carton labels to this day. Categories may then be used as a method for carcass fabrication and cut labeling based on these ciphers. An example of beef carcass categories and a carcass ticket has been provided in Figures 1 and 2, respectively.

Slaughter floor measures include hot standard carcass weight (**HSCW**, kg), P8 fat depth (mm, beef) or Grade Rule (**GR**) tissue depth (mm, sheep, measured at 110 mm from midline over 12th rib), and bruise score. Chiller assessment measures (eye muscle area, AUS-MEAT marbling, MSA marbling, sub-cutaneous rib fat, fat color, meat color, pH, hump height, and ossification) were incorporated over time to further define purchaser specifications and together formed the basis for beef grading (AUS-MEAT, 2018). Today, the AUS-MEAT language is used for the description of beef and lamb carcasses and marketing of cuts for sale nationally and internationally (AUS-MEAT, 2018, 2022).

### Meat Standards Australia grading

#### Beef-eating quality.

**Figure 3.**
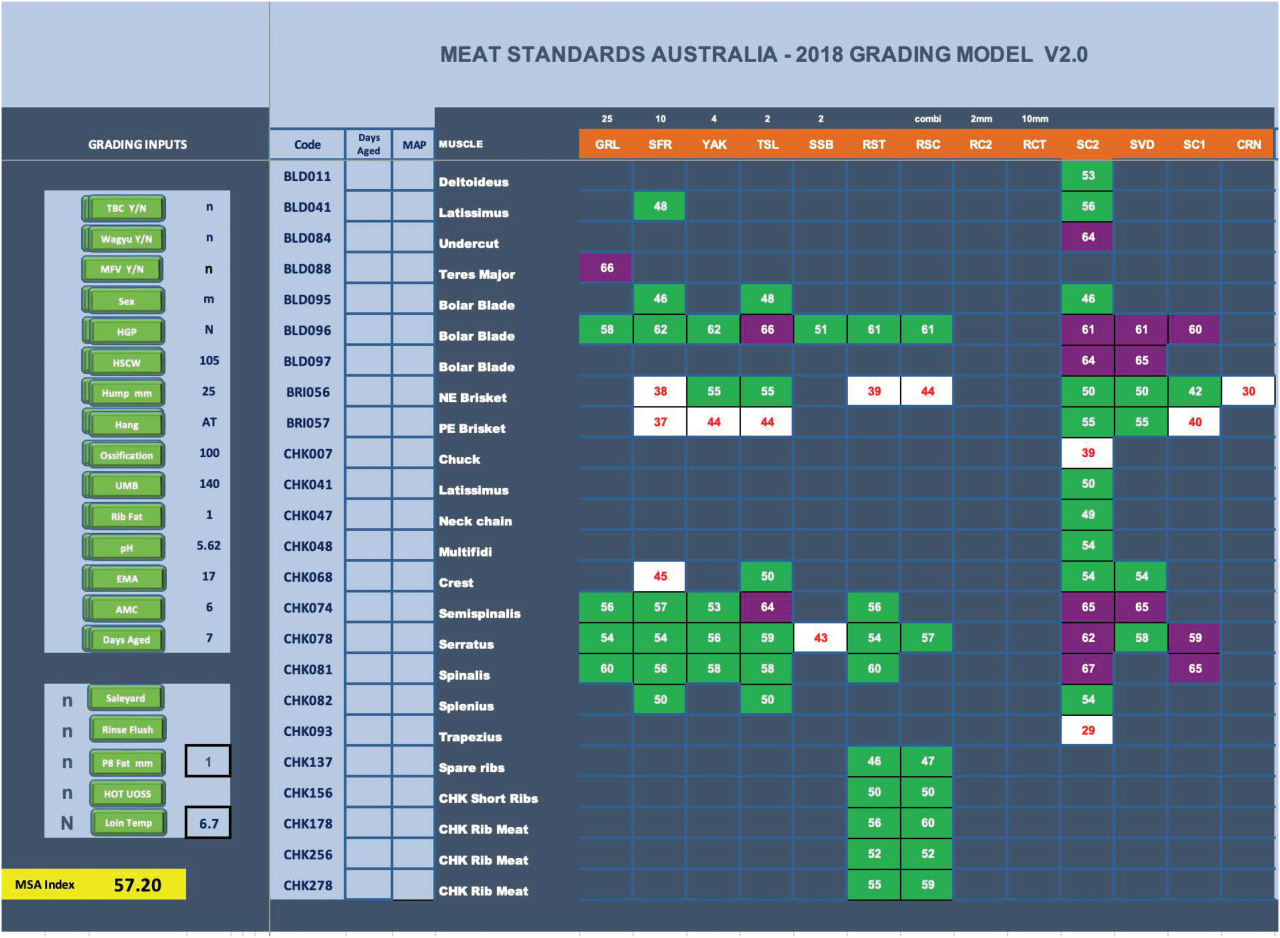
A snapshot example output from the beef Meat Standards Australia (MSA) cuts-based model, indicating grading input predictors, cut codes and muscle descriptions, cooking methods and predicted meat quality score of an individual carcass. See https://www.mla.com.au/marketing-beef-and-lamb/meat-standards-australia/msa-beef/ for the tips and tools kit for more detailed explanation of the grading inputs. 1. Grading input predictors on left side: TBC, tropical breed content; Wagyu, Wagyu breed classification (still under research and currently not used); MFV, milk fed vealer classification; Sex, sex (steer, heifer); HGP, hormone growth promotant status; HSCW, hot standard carcass weight; Hump, hump height; Hang, hanging technique; Ossification, ossification score; UMB, MSA marbling score; Rib Fat, rib fat depth; pH, ultimate pH; EMA, eye muscle area (not a predictor); AMC, AUS-MEAT color (not a predictor); Days Aged, days aging; Saleyard, selling method; Rinse Flush, vascular infusion post slaughter; P8 Fat, fat depth at the P8 site (Industry fat measure); HOT UOSS, ossification measured on the hot carcass; Loin Temp, loin temperature. 2. Cut codes and muscle description next to the grading inputs. MAP, modified high oxygen (80%) packaging. 3. Cooking methods shown on top of chart in orange boxes. GRL, grill; SFR, stir fry; YAK, yakiniku; TSL, thin slice; SSB, shabu shabu; RST, oven roast; RSC, combi oven roast; RC2, oven roast thin slice 2 mm; RCT, roast slice cold 10 mm; SC2, slow cook stew 2 hours; SVD, Sous Vide; SC1, slow cook stew 1 hour; CRN, corning infusion. 4. Body of table. White, ungraded; green, MSA 3 star every day quality; purple, MSA 4 star better than every day quality; orange, MSA 5 star premium quality orange (not present in current example); blank cells represent muscle by cooking combinations that have not been tested. Numbers represent the predicted MQ4 score.

The MSA grading program predicts consumer eating quality by grade, aging requirements, and cooking method across individual muscles. This is achieved by carcass grading inputs predicting the eating quality outcome of different cuts/muscles by different cooking methods as illustrated in Figure 3 (Watson et al., 2008c). The model produces a combined meat quality score (obtained from the consumer evaluations of tenderness, juiciness, flavor liking, and overall liking) between 0 and 100 for each muscle by cooking method by days aging (Figure 3; Watson et al., 2008c). Then based on a single palatability score, specifications for individual processors and/or retailer brands can be established by nominating minimum and maximum scores for each relevant cut.

MSA was introduced as a concept in the late 1980s due to concerns over declining beef prices and consumer feedback that Australian beef was inconsistent and often lower than the desired quality. Research to reduce such variability, improve consumer satisfaction and confidence, and develop the ability to predict the quality of a cooked meal as determined by consumers were embedded as high priorities in the 1995 beef industry strategic plan (Polkinghorne et al., 2008).

Today, MSA is a voluntary grading system for the Australian beef industry with adoption increasing to over 3.3 million head (54% of the national adult beef slaughter) per year (Meat and Livestock Australia, 2023). The remaining nongraded carcasses are mostly either cast for age cows or from cattle that are ineligible under current MSA guidelines largely due to extended time from farm to slaughter (>48 h) or intact males. The adoption rate is related to increased returns relative to ungraded product and the use of the system to support 194 company brands with a consistent eating quality guarantee. The MSA program was estimated to have delivered $259 million of additional farm gate revenue for beef producers in the 2022-2023 period (Meat and Livestock Australia, 2023).

Currently, over 140,000 Australian consumers have each evaluated seven samples of beef to provide the linkage between animal, processing, cut, and cooking interactions which underpin the current MSA prediction model. The MSA tasting protocols have previously been described by Watson et al. (2008a). Further collaborative and independent work utilizing identical protocols has added a further ~60,000 consumers from 12 countries (New Zealand, Korea, USA, France, Japan, Poland, South Africa, Northern Ireland, Wales, Irish Republic, England, and United Arab Emirates). The use of untrained consumer populations has been a critical success factor in the development of the MSA model as they (i) represent the true population who purchase beef and lamb products, (ii) allow for large numbers of meat samples to be evaluated, and (iii) provide meaningful qualitative estimates of consumer appeal aquired under standardized rigid preparation and sensory testing protocols (Watson et al., 2008a).

The current MSA beef model adjusts muscle eating quality outcomes for alternate carcass suspension methods and utilizes inputs from the AUS-MEAT chiller assessment language, including HSCW and ossification, as well as sex, pH, MSA marbling, hump height, sub-cutaneous rib fat, and hormone growth promotant status (Figure 3). These inputs have been identified through extensive research and consumer sensory taste panels to cause variation in consumer eating quality (Watson et al., 2008c). The weightings and interactions between these inputs differ by muscle and cooking method and are adjusted for days aging postmortem to predict the final eating quality score (Watson et al., 2008b, 2008c). As such the MSA model enables these inputs to be accounted for in the eating quality prediction and provides a system whereby individual cuts from cattle from a diverse range of production systems are valued, rather than simply being traded as commodity beef. Today the beef MSA model has expanded to over 300 cut by cooking method predictions with a snapshot provided in Figure 3.

Within the last eight years, changes to the MSA beef model included the removal of AUS-MEAT meat color and *Bos indicus* content (%). The *B. indicus* content % trait was initially assessed by the producer at consignment but has now been superseded by the measurement of carcass hump height (mm) in relation to HSCW and sex. A simple and independent method for assessing the *B. indicus* content of cattle was important to account for the higher frequency of the calpain-system gene markers that result in reduced tenderness (Robinson et al., 2012).

Meat color was initially retained in the MSA beef model as a screening trait due to the reported importance to consumers and association of meat colour with fresh, tender beef. However, research encompassing chiller and retail display colour assessment (both grading and objective measurements), consumer visual assessment, and taste panels found no relationship between meat colour and eating quality (Polkinghorne et al., 2017). Therefore, AUS-MEAT meat colour was removed from the model as a threshold variable, and pH cutoffs (>5.7) was retained as the indicator of high ultimate pH and dark-cutting beef (Thompson, 2016).

Additionally, high oxygen-modified atmosphere packaged cuts are also discounted at retail due to the detrimental impact of this packaging type on eating quality (Geesink et al., 2015; Frank et al., 2017).

#### Sheepmeat eating quality.

The same principles from beef MSA have been utilized to develop an MSA sheepmeat program which is now transitioning from a pathways model to a cuts-based prediction model. The lamb model development has utilized research flocks to provide comprehensive phenotypic and genotypic data, combined with untrained consumer sensory results (Pethick et al., 2005, 2006; Thompson et al., 2005; Fogarty et al., 2007; Van der Werf et al., 2010; Pannier et al., 2014, 2018).

Currently, the sheepmeat MSA program is commercially deployed as a pathways system. It comprises best practice guidelines for animal feeding, handling and curfew management, slaughter protocols to manage pH decline, and product aging and retail presentation of lamb cuts (Pethick et al., 2005; Young et al., 2005) to which all sectors along the supply chain (producer through to retail) must comply with. All sheepmeat MSA requirements and guidelines are publicly available (www.mla.com.au). The pathways system has resulted in improved lamb tenderness for consumers (Pethick et al., 2006); however, several limitations to the program remain because it is a mob-based “in or out” system. Until recently there has been no opportunity for individual carcass grading due to the lack of objective measures that can be operated at chain speed. Therefore, a commercial cut by cooking method prediction model (like for beef) is not in place for lamb. However, over the last 10 years, there has been increased research and investment into the development and adoption of objective devices to measure carcass traits that influence eating quality (Gardner et al., 2021b). This has highlighted that individual carcass grading in lamb is possible with commercial uptake of devices in Australian processing plants taking place. Additionally, ongoing consumer research has identified that a eating quality prediction model can be developed through the inclusion of measures like HSCW, LMY, and intramuscular fat and meat aging time (Mortimer et al., 2014; Pannier et al., 2014).

Accordingly, a new cuts-based MSA sheepmeat model has been developed and the commercialization of the model is currently underway (Meat and Livestock Australia, 2023). The model has been expanded from a pathways model into an individual carcass grading model, with traits of HSCW, LMY, intramuscular fat, and days aging (Pannier et al., 2018), and includes eight cuts (loin, rump, topside, knuckle, outside, easy carve leg, rack, and shoulder) and two cooking methods (grilling and roasting) to form nine cut by cooking method combinations. Once fully commercially implemented this model would enable sheepmeat eating quality grades to be defined for good everyday (3 stars), better than everyday (4 stars), and premium (5 stars) quality, aligning with the existing beef MSA quality grades. This will add value to the Australian sheepmeat industry by enabling the development of branded lamb products, underpinned by eating quality. In conjunction with corresponding price points, branded products will give consumers greater product choice and deliver increased value through their willingness to pay for higher-quality products (Tighe et al., 2017). This is expected to apply to both domestic and international consumers, with recent studies indicating that an Australian sheepmeat eating quality model can be used to predicted eating quality in a wide range of international consumer groups (O’Reilly et al., 2020, 2023) from China and the USA (Thompson et al., 2005).

### Carcass yield

**Figure 4.**
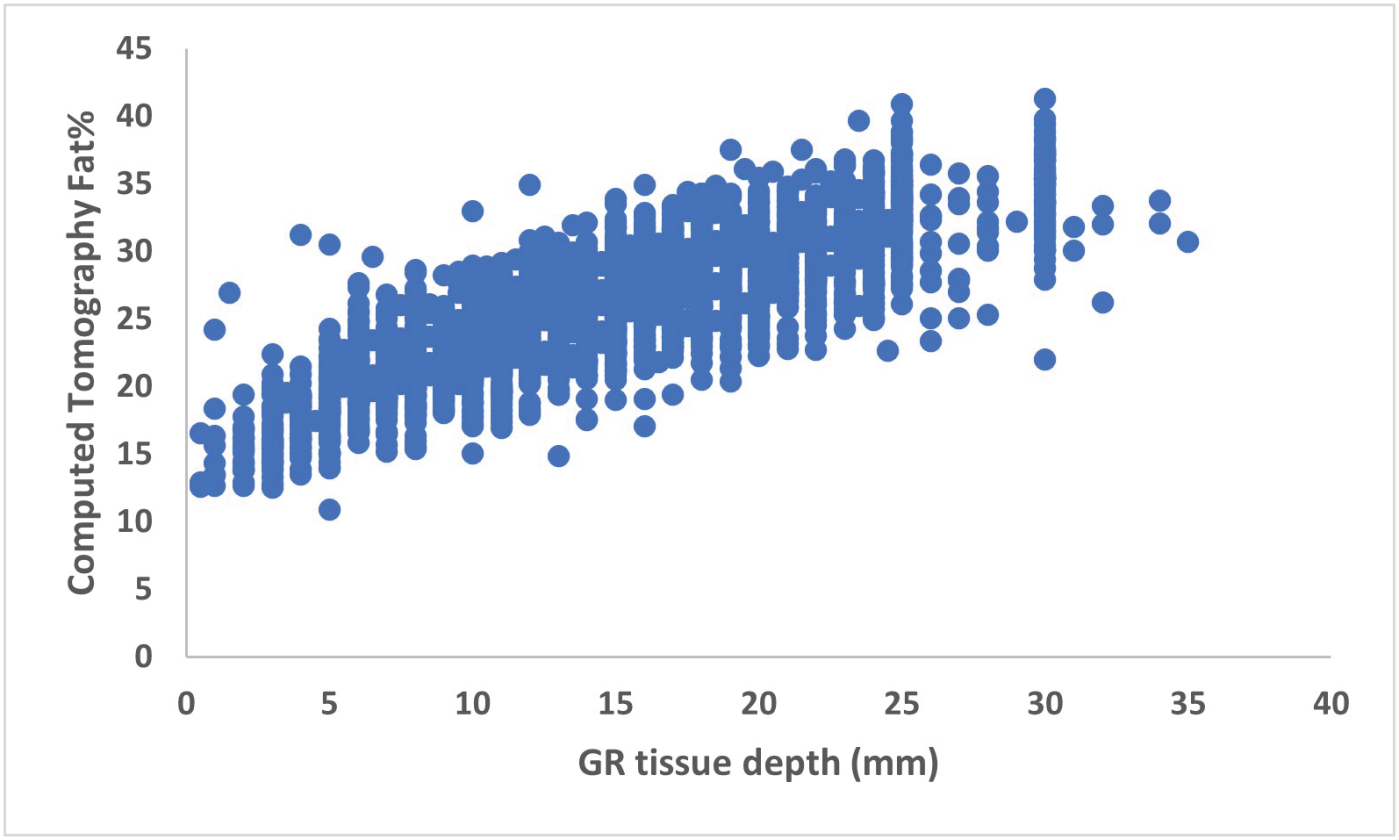
Prediction of computed tomography carcass fat percentage using GR tissue depth in lamb carcasses (*n* = 3,750). Courtesy by A. Williams.

Carcass grading for LMY at some level is a common theme for all grading schemes around the world. However, the methods used to assess it are highly variable with carcass weight, shape, fat thickness/abundance at various locations, and eye muscle area among the common parameters utilised (Hocquette et al., 2022). In Australia, HSCW combined with fat depth at the P8 site in cattle or tissue depth at the GR site in lamb underpins payment grids. However, these methods are very crude and inaccurate when compared to the “Gold Standard” computer tomography determined carcass composition in beef and lamb (Williams et al., 2017a, 2017b) (Figure 4). Furthermore, although a key driver of cut weight is carcass weight, carcasses of the same weight can differ greatly in the weight of their saleable cuts dissected from them. This is due to variation in fatness, with fatter carcasses yielding smaller commercial cut weights (Gardner et al., 2021a).

Thus, the role of LMY has a close association to saleable yield, especially for boned product, in determining value as it defines the final amount of saleable meat available for human consumption. However, the value of the meat ($/kg) is also critical as reported by McGilchrist et al. (2022) whom showed that beef brands selling all cuts with MSA eating quality premiums had up to 70% of the relative carcass value attributed to eating quality. Not surprisingly if a “flat-price” was applied across all cuts, 97% of the value was determined by LMY (McGilchrist et al., 2022). Therefore, LMY is clearly an important parameter for value-based trading regardless of branding structures and more accurate methods to measure LMY are required (Pethick et al., 2021).

### Emerging objective measurement technologies in Australia

In recent years, the industry has seen the emergence of new LMY and eating quality technologies with the aim to provide increased objectivity and repeatability for grading (Gardner et al., 2021b). This has occurred through investment by government and industry via the Advanced Livestock Measurement Technologies (ALMTech) Project. This project has enabled the development of new technologies to objectively measure LMY and eating quality grading traits (Gardner et al., 2021b) and facilitated the commercialization of devices for use in the beef and sheep industries.

**Figure 5.**
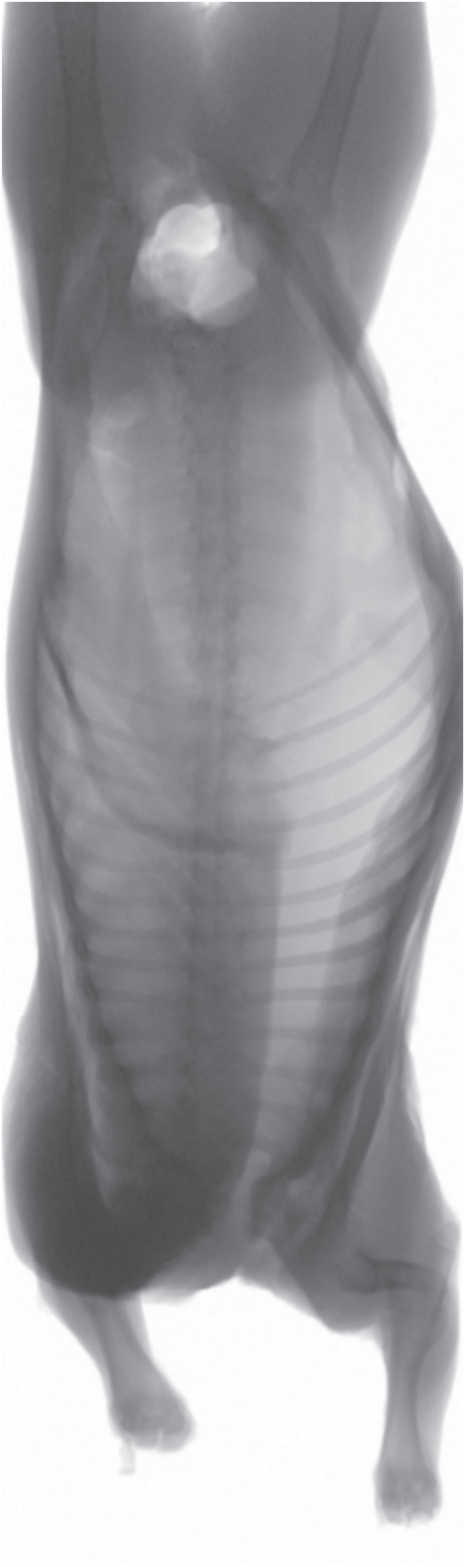
DEXA image generated of lamb carcass for lean meat yield prediction. Courtesy by G.E. Gardner.

Technologies have been developed that measure beef and lamb whole carcass composition and LMY including rapid dual x-ray absorptiometry (**DEXA**, Figure 5; Connaughton et al., 2020; Calnan et al., 2021; Gardner et al., 2021a) and 3D imaging (Alempijevic et al., 2021) or single-site point technologies such as microwave probes (Marimuthu et al., 2021a, 2021b,) which measure GR tissue depth and P8 fat depth in live animals and carcasses. These systems are also being used to predict cut weights prior to boning (Gardner et al., 2021a), creating the opportunity to optimize carcass fabrication matched to market specification.

Multiple technologies have also been developed for quality grading (especially marbling and/or intramuscular fat) in beef and lamb including the multispectral and RGB vision cameras (Stewart et al., 2021a, 2021b), DEXA (Anderson et al., 2021), optical coherence tomography, fiber optic and near-infrared probes (Fowler et al., 2020), and hyperspectral cameras (Dixit et al., 2021; Hitchman et al., 2021). Many of these technologies have been accredited by AUS-MEAT (https://www.ausmeat.com.au/services/list/meat/equipment-approval-information/) to measure carcass traits and are in the process of being adopted by supply chains across Australia.

#### Challenges and opportunities.

It is debatable whether subjective traits should be used to train and validate objective technologies (Ferguson, 2004; Jackman et al., 2009). Whilst a moderate level of precision and accuracy can be achieved by training and validating technologies against human graders, for any technology, the level of performance is constrained by the measurement error in calibration. Thus, while devices can be biased because of the initial settings and sensitivity of the technology, validation precision and accuracy are determined by the initial calibration to the reference data set by expert graders (Moore et al., 2010; Stewart et al., 2021b).

A critical point is that the use of expert grader panels to calibrate and validate technologies does not address inherent bias of graders (Jang et al., 2017) or future proof the industry for new devices, due to the use of different grader panels over time. In addition, the use of graders limits the ability to describe more variation in LMY and eating quality through identification of new traits, for example, tenderness and fatty acid profile which may only be measured by technologies. Moreover, in the sheep industry, carcasses are not ribbed, limiting the use of graders to train devices for eating quality traits.

Thus, the use of objective traits (such as chemical intramuscular fat percentage) should be considered as the industry standard to which technologies are compared and accredited. Importantly, chemical intramuscular fat percentage and LMY% have recently been approved as traits in beef and lamb (https://www.ausmeat.com.au/services/list/meat/equipment-approval-information/) signaling the importance that the industry places on objective grading and future proofing the accreditation of technologies for the improvement of grading.

## Carcass Value: Opportunities and Challenges in the Australian Red Meat Industry

As previously described, beef and lamb carcasses are mostly traded on payment grids based on carcass weight and P8 fat depth or GR tissue depth, dentition, and sex. These grids typically have relatively wide windows for weights and fatness (Pitchford et al., 2021) and offer little incentive for precise slaughter targets that enable livestock producers to optimize production systems which maximize financial and biological efficiency. For many beef processors, an additional overlay of eating quality is applied to payment grids which enables segregation of cuts into MSA quality grades that then underpins brands (Pethick et al., 2021). However, in most cases, MSA is still underutilized and simply applied as an in-or-out system (3 stars and above) or includes an AUS-MEAT marbling grid (e.g., 2+, 5+, and 7+) that is applied to a few high-value cuts, typically in the long-fed or wagyu markets.

Whilst this system may be applied on an individual carcass level if traded “over-the-hooks”, payment grids as described prevent true quantification of carcass value for the producer, processor, retailer, and consumer. The issue with the current “language-derived” grid system is that it cannot accurately describe true carcass value, which is simply defined as the weight of cuts (yield) multiplied by the eating quality of the cuts within a carcass (Pethick et al., 2021; McGilchrist et al., 2022).

The eating quality component of carcass value is well established in Australia, through the MSA grading system. Regarding consumer value, MSA grading enables the prediction of consumers purchasing behavior and the ability to differentiate value relative to carcass quality and meal satisfaction. Via MSA consumer willingness to pay data it is known that increasing eating quality from 2 stars (unsatisfactory) to 5 stars (premium) attracts increased retail prices by 2- to 3-fold (Lyford et al., 2010; Tighe et al., 2017).

Producers can also use MSA to understand the carcass value. In a recent study, a carcass index was proposed which comprised a weighted average of the different MSA grades across 39 muscles in the carcass (McGilchrist et al., 2019). The MSA index was initially proposed as a tool for producers and industry to benchmark the impact of management and genetic factors on overall eating quality of the carcass (McGilchrist et al., 2019). The MSA index has also been used in combination with retail beef yield data to differentiate carcass value models (Pitchford et al., 2021; McGilchrist et al., 2022).

However, it is the application of cuts/muscle-based eating quality in conjunction with the weight of the cuts/muscle that will enable transparent carcass valuation. This has the transformational potential to move livestock and meat trading based on averages and poor estimates to a transparent value-based platform. True carcass value underpins value-based marketing and links carcass price and value to important traits for the consumer and processor. This system removes unreliable yield and quality traits and importantly uses continuous pricing functions to improve market signals to livestock suppliers (Cross and Savell, 1994; Johnson and Ward, 2005).

Predicting the weight of commercial cuts from entire carcasses prior to manufacturing is valuable as it enables processors to sort carcasses prefabrication and optimize cut plans. This can maximize the weight of saleable meat into the highest value markets which increases profit and minimizes carcass trimming. It also allows processors to establish boning room benchmarking systems to monitor predicted and actual yields, which underpin carcass value and more accurate pricing grids. For retailers, predicting carcass cut weights prior to boning enables accurate allocation of carcasses to product orders, with more uniform cut size, weight and price for packaging (e.g., tray size), and retail display, which may influence consumer appeal and purchasing decisions (Gardner et al., 2021a).

In addition to carcass yield and eating quality, animal health is another emerging area identified as a significant driver of carcass value either directly, based on offal products, or indirectly through animal health issues driving reduced LMY and eating quality. Several processors have incorporated animal health data (e.g., offal condemnation) into feedback systems and payment grids. This can provide opportunities for farm assurance programs, which may deliver financial incentives and assist producers manage on-farm animal health issues (Guy et al., 2018).

Value-based marketing underpinned by objective yield, eating quality, and consumer data creates a more transparent method for producers to understand carcass value relative to the processor and its brands/markets. This enables producers to make better decisions regarding genetic improvement and on-farm management and productivity (Walkom et al., 2021). For processors, it facilitates optimized carcass fabrication to match market specification whilst reducing wastage and enables the development of benchmarking systems and builds producer trust. However, commercial adoption of value-based marketing in Australia is challenging, given the diversity of carcass phenotypes and the marketing options available. Moreover, under most fabrication systems, the traceability of carcass identification to primal is lost, which limits extracting the full value from cuts-based grading (Doljanin et al., 2016). Carcass sorting to cut quality thresholds can mitigate this to a degree, but this limits how many cuts can be value added. Therefore, individual supply chains are required to assess the relative importance of eating quality and LMY in their business and supply chains in conjunction with marketing opportunities. A “one-size-fits-all” model will not work, thus value-based marketing systems will be processor dependent and require industry support.

## Conclusion

Australian carcass and meat evaluation has evolved from very basic and imprecise assessments to a sophisticated system that incorporates eating quality-based carcass grading. However, further investment and research are required to improve carcass yield grading. The emergence of objective measurement technologies has the potential to transform the Australian red meat industry, providing precise and accurate measurement of carcass quality and yield which are critical to the commercial application of value-based marketing schemes.

## About the Authors

**Sarah Stewart** is a research fellow in Meat Science based in Perth, working in the School of Agricultural Sciences at Murdoch University, Western Australia. She completed her PhD studies at Murdoch which investigated the impact of preslaughter stress on intermediary metabolism and meat quality in lamb. In her current role, she manages the eating quality program for the Advanced Livestock Measurement Technologies (ALMTech) project. Her research has contributed to the development and commercialization of objective technologies for grading quality traits in beef, lamb, and pork. She is also a keen participant at an industry level, with involvement across several industry supply chain working and technical groups. In addition, she is a member of the Australian Intercollegiate Meat Judging (ICMJ) Committee, where she currently holds the position of sponsorship coordinator and has held previous coaching positions including the Australian National and Murdoch University teams. **Corresponding author:**s.stewart@murdoch.edu.au

**Rod Polkinghorne** graduated from Dookie Agricultural College with a Diploma of Agricultural Science (Hons). He was awarded an honorary Doctorate by Murdoch University in recognition of his scientific contribution to meat science and is an adjunct professor at the University of New England. He has enjoyed an extensive industry career in beef and dairy feedlotting, breeding, product development, and retailing. His research work has focused on utilizing untrained consumer sensory evaluation to quantify the impact and interaction of animal production, processing, carcase measures, and cooking on individual muscle-cooked performance. The Meat Standards Australia program was the outcome of this work overseen by the MSA Pathways Committee which he chairs. He works extensively with universities, industry bodies, and commercial companies to evaluate local beef production systems and guide practical commercial application of the science to deliver effective beef branding and associated grading in multiple countries.

**David Pethick** is a professor emeritus in Animal Production and Science working in the School of Agricultural Sciences at Murdoch University, Western Australia. He completed PhD studies at Cambridge University in 1980 investigating the metabolism of volatile fatty acids, glucose, and long-chain fatty acids in the tissues of sheep. Subsequently, a major area of work has been to understand how to improve and guarantee the consumer acceptance of cooked beef and lamb. This includes working across the “value chain” (on farm, livestock, transport, abattoir, and retail) to underpin lamb and beef quality. Specific contributions include the nutritional and stress management required to deliver high levels of muscle glycogen, intramuscular fat, and tenderness to underpin beef and lamb brands. His work has contributed to the development of the Meat Standards Australia scheme which is used by the Australian Industry to underpin the delivery of quality beef and lamb to consumers.

**Liselotte Pannier** is an associate professor in Animal Production and Science working in the School of Agricultural Sciences at Murdoch University, Western Australia. She completed a PhD in Ireland, through the University College of Dublin and the Ashtown Food Research Centre (Teagasc), focusing on genetic markers in beef meat quality traits. Since then, she has been a leading researcher at Murdoch University focusing on the impact of production and genetic factors on consumer meat eating quality and preferences of domestic and international consumers. Her work entails looking at aspects of animal age, feeding types, different cuts, and sensory cooking methods in relation to consumer eating quality, as well as understanding the impact of carcass traits, muscularity, and lean meat yield on eating quality. Development of the Meat Standards Australia sheepmeat eating quality prediction model with inclusion of objective carcass measures is also key to her research.
